# The effects of magnesium and vitamin E co-supplementation on parameters of glucose homeostasis and lipid profiles in patients with gestational diabetes

**DOI:** 10.1186/s12944-018-0814-5

**Published:** 2018-07-20

**Authors:** Maryam Maktabi, Mehri Jamilian, Elaheh Amirani, Maryam Chamani, Zatollah Asemi

**Affiliations:** 1 Endocrinology and Metabolism Research Center, Department of Gynecology and Obstetrics, School of Medicine, Arak University of Medical Sciences, Arak, Iran; 2 Research Center for Biochemistry and Nutrition in Metabolic Diseases, Kashan University of Medical Sciences, Kashan, Iran; 3 Department of Gynecology and Obstetrics, School of Medicine, Iran University of Medical Sciences, Tehran, Iran

**Keywords:** Supplementation, Insulin, Lipid, Gestational diabetes

## Abstract

**Background:**

Magnesium and vitamin E are known to exert multiple beneficial effects, such as anti-glycemic and anti-lipidemic properties. The aim of this study was to determine the effects of magnesium and vitamin E co-supplementation on metabolic status of women with gestational diabetes (GDM).

**Methods:**

This randomized, double-blinded, placebo-controlled trial was conducted among 60 subjects diagnosed with GDM, aged 18–40 years. Subjects were randomly allocated into two groups to receive 250 mg/day magnesium oxide plus 400 IU/day vitamin E supplements or placebo (*n* = 30 each group) for 6 weeks. Participants’ blood samples were taken to determine their metabolic profiles.

**Results:**

Subjects who received magnesium plus vitamin E supplements had significantly lower fasting plasma glucose (β − 5.20 mg/dL; 95% CI, − 7.88, − 2.52; *P* = 0.002), serum insulin levels (β − 2.93 μIU/mL; 95% CI, − 5.68, − 0.18; *P* = 0.02) and homeostasis model of assessment-insulin resistance (β − 0.78; 95% CI, − 1.42, − 0.14; *P* = 0.01), and higher quantitative insulin sensitivity check index (β 0.01; 95% CI, 0.005, 0.02; *P* = 0.002) compared with placebo. In addition, magnesium plus vitamin E supplementation resulted in a significant reduction in serum triglycerides (β − 50.31 mg/dL; 95% CI, − 67.58, − 33.04; *P* < 0.001), VLDL- (β − 10.06 mg/dL; 95% CI, − 13.51, − 6.60; *P* < 0.001), total- (β − 26.10 mg/dL; 95% CI, − 41.88, − 10.33; *P* = 0.004), LDL- (β − 15.20 mg/dL; 95% CI, − 29.50, − 0.91; *P* = 0.03) and total-/HDL-cholesterol ratio (β − 0.46; 95% CI, − 0.72, − 0.19; *P* < 0.001) compared with placebo. Magnesium and vitamin E co-supplementation did not affect HDL-cholesterol levels.

**Conclusions:**

Overall, magnesium and vitamin E co-supplementation for 6 weeks in women with GDM significantly improved glycemic control and lipid profiles, except for HDL-cholesterol levels.

**Clinical trial registration number:**

http://www.irct.ir: IRCT20170513033941N24.

## Background

Gestational diabetes mellitus (GDM) is defined as carbohydrates intolerance which first recognized at second or third trimester of pregnancy and has reported to increasing worldwide [1]. It influences approximately 1 to 20% of all pregnancies worldwide as well as its prevalence among Iranian women is about 4 to 9% of all pregnancies [2]. Both environmental risk factors and genetic background contribute to the development of GDM [3]. In addition to maternal and fetal complications, GDM is associated with the elevated potential for metabolic disorder including type 2 diabetes mellitus (T2DM) and cardiovascular disease (CVD) in future life of both mother and offspring [4, 5]. In addition, insulin resistance and dyslipidemia are the hallmarks of GDM [6, 7].

There is evidence demonstrating that magnesium is required more during pregnancy [8]. In addition, hypomagnesemia might lead to impaired glucose tolerance [9]. Few studies have reported low circulating levels of magnesium and vitamin E in women with GDM [10, 11]. Furthermore, several human studies have demonstrated the beneficial effects of single magnesium [12] or vitamin E supplementation on metabolic profiles. In a meta-analysis, magnesium supplementation resulted in a significant reduction in insulin resistance, but did not affect fasting glucose and insulin levels [13]. Earlier, we have shown that magnesium supplementation for 6 weeks to women with GDM led to a significant reduction in triglycerides and VLDL-cholesterol levels, but did not affect other lipid profiles [14]. In addition, some studies have reported the beneficial effects of vitamin E supplementation on glucose metabolism and lipid profiles in patients with metabolic syndrome [15, 16]. Recently, it has been suggested that joint magnesium and vitamin E supplementation in diabetic rat was more beneficial to improve lipid profiles and blood viscosity rather than magnesium alone [17]. In another study, combined vitamin E and magnesium supplementation could effectively improve triglycerides levels of obese rats, better than vitamin E alone [18].

This evidence might suggest the importance of magnesium and vitamin E co-supplementation on metabolic profiles in women with GDM. According to our best knowledge, data on the effects of magnesium and vitamin E co-supplementation on metabolic status of patients with GDM are scarce. Therefore, the aim of this study was to evaluate the effects of magnesium and vitamin E co-supplementation on metabolic status of patients with GDM.

## Methods

### Participants

This randomized, double-blinded, placebo controlled clinical trial, registered in the Iranian website for registration of clinical trials (no: IRCT20170513033941N24), was conducted among sixty women with GDM, aged 18–40 years and non-diabetic before pregnancy, diagnosed using American Diabetes Association guidelines [19] from December 2017 through March 2018. The study was approved by the ethics committee of Arak University of Medical Sciences (AUMS) and written informed consent was taken from all participants prior to the commencement of the study. Exclusion criteria were; taking magnesium and vitamin E supplements 3 months before the intervention insulin therapy required during the intervention, experiencing pre-eclampsia, eclampsia, hypo and hyperthyroidism, and being smokers.

### Study design

To decrease the effects of potential confounders, stratified randomization was performed at the beginning of the study for all participants according to age and BMI. Then, participants in each block were randomly allocated into two treatment groups to take either 250 mg/day magnesium oxide (twenty-first Century, Arizona, USA) and 400 IU/day vitamin E (Zahravi, Tabriz, Iran) or placebo (Barij Essence, Kashan, Iran) (*n* = 30 each group) for 6 weeks. Randomization assignment was conducted using computer-generated random numbers. Randomization and allocation concealment for both researchers and participants were carried out by a trained staff at the gynecology clinic. Compliance to the magnesium and vitamin E intake was assessed through measuring serum magnesium levels. The consumption of magnesium supplements and placebos during the study was also checked by asking subjects to return the medication containers back and receiving brief daily cell phone reminders to take the supplements. All patients were advised to maintain their routine dietary habits without any changes in their other lifestyle factors such as physical activity during the study. All patients completed 3-day food records and three physical activity records presented as metabolic equivalents (METs) at weeks 0, 3, 6 of the treatment.

### Assessment of anthropometric measures

Participants’ weight and height were measured using a standard scale (Seca, Hamburg, Germany) in a fasting status at baseline and after 6-weeks’ intervention. Body mass index (BMI) was calculated as weight in kg divided by height in meters squared.

### Assessment of outcomes

In this study, glycemic control was considered as the primary outcome, and lipid profiles the secondary outcomes.

### Biochemical assessment

10 ml fasting blood samples were collected from participants at weeks 0 and 6 of the intervention. Commercial kits were used to measure serum magnesium, fasting plasma glucose (FPG), serum triglycerides, total-, VLDL-, LDL- and HDL-cholesterol concentrations (Pars Azmun, Tehran, Iran). Serum magnesium levels were measured by enzymatic method. The inter- and intra-assay coefficient variances (CVs) for magnesium, FPG, lipid profiles measurements were less than 5%. Serum insulin values were assessed using an ELISA kit (Monobind, California, USA) with the intra- and inter-assay CVs of lower than 6%. The homeostatic model of assessment for insulin resistance (HOMA-IR) and the quantitative insulin sensitivity check index (QUICKI) were determined according to suggested formulas [20]. HOMA-IR was calculated according to the following formula: fasting insulin (μIU/mL) x fasting glucose (mmol/L)/22.5 [20]. QUICKI was calculated as QUICKI = 1/[log(I_0_) + log(G_0_)], where I_0_ is the fasting insulin, and G_0_ is the fasting glucose [20].

### Sample size

Type one (α) and type two errors (β) were defined as 0.05, and 0.20, respectively to have the study power of 80%. Based on a previous published study [14], we used 1.05 as the mean difference of the HOMA-IR and 1.30 as SD. Calculating sample size, we required 25 patients in each treatment group; allowing for 20% dropouts in each group, the final sample size was considered to be 30 patients per treatment group.

### Statistical analyses

Kolmogorov-Smirnov test was done to determine the normality of data. To detect the differences in anthropometric measures and dietary intakes between treatment groups, we used independent-samples *t*-test. To determine the effects of magnesium and vitamin E co-supplementation on parameters of glucose homeostasis and lipid profiles, we used general linear model and one-way repeated measures analysis of variance. In this analysis, treatment variable (magnesium plus vitamin E vs. placebo) was regarded as between-subject factor and time-points (baseline and week 6 of intervention) as within-subject factor. The effect sizes were presented as the mean differences with 95% confidence intervals. *P*-values < 0.05 were considered statistically significant. All statistical analyses were done using the Statistical Package for Social Science version 18 (SPSS Inc., Chicago, Illinois, USA).

## Results

**Fig. 1:**
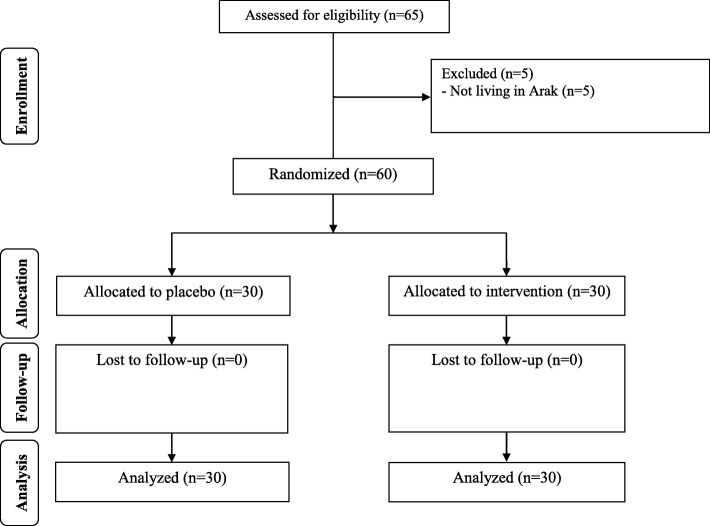
Summary of patient flow diagram

During the enrollment phase of the study, 65 women with GDM were invited to participate in the trial; however, 5 participants were excluded from the study, due to not meeting the inclusion criteria. Finally, 60 participants [placebo (*n* = 30) and magnesium plus vitamin E (n = 30)] completed the trial (Fig. 1).

Mean age, height, weight and BMI at baseline and after the 6-week treatment were not statistically different between the two groups (Table 1).

**Table 1:** General characteristics of study participants

	Placebo group (*n* = 30)	Magnesium plus vitamin E group (*n* = 30)	P^1^
Age (y)	31.5 ± 3.2	30.1 ± 5.9	0.25
Height (cm)	162.3 ± 4.5	162.5 ± 5.1	0.89
Weight at study baseline (kg)	73.7 ± 7.7	72.7 ± 12.6	0.74
Weight at end-of-trial (kg)	75.1 ± 7.7	74.1 ± 12.5	0.70
Weight change (kg)	1.4 ± 0.4	1.3 ± 0.5	0.33
BMI at study baseline (kg/m^2^)	28.0 ± 3.1	27.6 ± 4.7	0.68
BMI at end-of-trial (kg/m^2^)	28.5 ± 3.1	28.0 ± 4.7	0.64
BMI change (kg/m^2^)	0.5 ± 0.2	0.5 ± 0.2	0.29

Data are means± standard deviation

^1^ Obtained from independent sample *t*-test

Using 3-day dietary records, obtained during the intervention, there was no statistically significant difference in terms of dietary macro- and micro-nutrient intakes between magnesium plus vitamin E and placebo groups (Data not shown).

Subjects who received magnesium plus vitamin E supplements had significantly lower FPG (β − 5.20 mg/dL; 95% CI, − 7.88, − 2.52; *P* = 0.002), serum insulin levels (β − 2.93 μIU/mL; 95% CI, − 5.68, − 0.18; *P* = 0.02) and HOMA-IR (β − 0.78; 95% CI, − 1.42, − 0.14; *P* = 0.01), and higher QUICKI (β 0.01; 95% CI, 0.005, 0.02; *P* = 0.002) compared with placebo (Table 2). In addition, magnesium plus vitamin E supplementation resulted in a significant reduction in serum triglycerides (β − 50.31 mg/dL; 95% CI, − 67.58, − 33.04; *P* < 0.001), VLDL- (β − 10.06 mg/dL; 95% CI, − 13.51, − 6.60; *P* < 0.001), total- (β − 26.10 mg/dL; 95% CI, − 41.88, − 10.33; *P* = 0.004), LDL- (β − 15.20 mg/dL; 95% CI, − 29.50, − 0.91; *P* = 0.03) and total-/HDL-cholesterol ratio (β − 0.46; 95% CI, − 0.72, − 0.19; *P* < 0.001) rather than placebo group. Magnesium and vitamin E co-supplementation did not affect HDL-cholesterol levels.

**Table 2:** The effect of magnesium and vitamin E co-supplementation on metabolic status in women with gestational diabetes

Variables	Placebo group (n = 30)		Magnesium plus vitamin E group (*n* = 30)		Difference in outcome measures between intervention and placebo treatment groups^1^	
	Baseline	Week 6	Baseline	Week 6	β (95% CI)	*P* ^2^
Magnesium (mg/dL)	2.07 ± 0.15	2.02 ± 0.15	2.04 ± 0.22	2.18 ± 0.13	0.17 (0.11, 0.23)	< 0.001
FPG (mg/dL)	91.9 ± 5.6	91.8 ± 6.4	90.3 ± 6.2	85.4 ± 5.9	−5.20 (−7.88, −2.52)	0.002
Insulin (μIU/mL)	13.4 ± 4.1	14.9 ± 8.9	13.2 ± 3.6	11.5 ± 3.7	−2.93 (−5.68, −0.18)	0.02
HOMA-IR	3.0 ± 0.9	3.3 ± 2.0	3.0 ± 0.8	2.4 ± 0.8	−0.78 (−1.42, − 0.14)	0.01
QUICKI	0.32 ± 0.01	0.32 ± 0.02	0.32 ± 0.01	0.33 ± 0.02	0.01 (0.005, 0.02)	0.002
Triglycerides (mg/dL)	208.9 ± 52.1	54.2	206.3 ± 63.5	169.1 ± 65.9	−50.31 (−67.58, −33.04)	< 0.001
VLDL-cholesterol (mg/dL)	41.8 ± 10.4	44.5 ± 10.8	41.2 ± 12.7	33.8 ± 13.2	−10.06 (−13.51, −6.60)	< 0.001
Total cholesterol (mg/dL)	229.5 ± 41.2	232.9 ± 42.4	223.3 ± 49.0	201.3 ± 46.7	−26.10 (−41.88, − 10.33)	0.004
LDL-cholesterol (mg/dL)	130.3 ± 36.4	131.6 ± 39.0	127.9 ± 41.7	113.4 ± 45.5	−15.20 (−29.50, −0.91)	0.03
HDL-cholesterol (mg/dL)	57.4 ± 13.2	56.8 ± 11.3	54.1 ± 9.6	54.0 ± 8.7	0.70 (−3.07, 2.09)	0.68
Total-/HDL-cholesterol ratio	4.1 ± 1.0	4.2 ± 1.0	4.2 ± 0.8	3.8 ± 0.8	−0.46 (−0.72, − 0.19)	< 0.001

Data are mean ± SDs

^1^”Outcome measures” refers to the change in values of measures of interest between baseline and week 6. β [difference in the mean outcomes measures between treatment groups (magnesium plus vitamin E group = 1 and placebo group = 0)]

^2^ *P* values represent the time × group interaction (computed by analysis of the repeated measures ANOVA)

FPG, fasting plasma glucose; HOMA-IR, homeostasis model of assessment-insulin resistance; HDL-cholesterol, high density lipoprotein-cholesterol; LDL-cholesterol, low density lipoprotein-cholesterol; QUICKI, quantitative insulin sensitivity check index; VLDL-cholesterol, very low density lipoprotein-cholesterol

## Discussion

In the present study, we examined the effects of co-supplementation of magnesium and vitamin E on parameters of glucose homeostasis and lipid profiles in women with GDM. We found that magnesium and vitamin E co-supplementation to women with GDM for 6 weeks improved glycemic control and lipid concentrations except for HDL-cholesterol values.

Gestational diabetes mellitus occurs because of altered glucose metabolism and peripheral insulin resistance [21]. Our study indicated that magnesium and vitamin E co-administration to women with GDM lowered serum FPG, insulin levels and HOMA-IR, and led to a significant rise in QUICKI. Similar to our findings, a meta-analysis conducted by Simental-Mendia et al. [13] revealed that magnesium supplementation for at least 4 months improved FPG and HOMA-IR in both diabetic and non-diabetic individuals. In addition, our previous study indicated that a 6-week co-supplementation with magnesium and other nutrients in patients with GDM had beneficial effects on FPG, insulin levels, HOMA-IR and QUICKI [22]. Moreover, Rafraf et al. [23] reported that vitamin E supplementation to people with T2DM for 8 weeks reduced serum FPG levels. However, some researchers failed to find the beneficial effects of magnesium [24] or vitamin E supplementation on glycemic control. For instance, in a meta-analysis conducted by Xu et al. [25], vitamin E supplementation did not affect parameters of insulin metabolism. In addition, a 3-month magnesium supplementation in hypomagnesic pre-diabetic patients with chronic kidney disease did not change FPG levels [26].

Maternal insulin resistance in GDM might increase placental size, though decrease placental efficiency, subsequently might affect fetal growth [27]. It also has been demonstrated that well glycemic control may improve pregnancy outcomes [28]. Magnesium is involved in glucose metabolism through its effects on tyrosine kinase activity of the insulin receptors [29]. It also regulates glucose uptake via its influence on glucose transporte-4 activity [29], and regulates oxidative pathway of glucose metabolism through the activation of pyruvate dehydrogenas [30]. Furthermore, the beneficial effects of vitamin E intake on insulin resistance may be explained through its effect on suppressing oxidative stress and gene expression of peroxisome proliferator activated-receptor (PPAR) alpha [31]. Also, vitamin E can mediate glucose metabolism by stimulating glutathione and magnesium levels [32].

Gestational diabetes mellitus is accompanied by lipid profiles changes including an elevated triglyceride, total- and LDL-cholesterol levels [33]. In the present study, we observed that magnesium and vitamin E co-supplementation to women with GDM reduced triglycerides, VLDL-, total-, LDL- and total-/HDL-cholesterol, but did not affect HDL-cholesterol levels. In agreement with our findings, the results of another clinical study indicated that magnesium supplementation for 4 months decreased triglycerides levels in pre-diabetic patients with hypomagnesemia [34]. Consistent with our results, Ekhlasi et al. [35] reported that synbiotic and vitamin E co-supplementation reduced triglycerides, total- and LDL-cholesterol levels, but did not affect HDL-cholesterol concentrations. Moreover, a significant reduction in total cholesterol levels was reported after vitamin E supplementation to women with metabolic syndrome [36]. In contrast, in a meta-analysis conducted by Xu et al. [25] there was no significant effects of vitamin E supplementation on lipid profiles. On the other hand, magnesium supplementation could not improve lipid levels in both diabetic and non-diabetic individuals [37]. Hypertriglyceridemia during pregnancy worsen insulin resistance [38] and seems to be an independent predictor of fetal macrosomia [39]. Recent studies have reported a positive association between maternal triglycerides levels and the risk of large for gestational age neonate, independent of glycemic controls [40]. Magnesium acts as the cofactor of lipoprotein lipase which induces chylomicrone clearance and delays postprandial increase in triglycerides levels [41]. Vitamin E, beside its antioxidant effects, modulates gene expression of PPAR gama, which involves in lipid metabolism [42].

Our study had a few limitations. Due to lack of enough funding, we could not evaluate the effects of magnesium and vitamin E co-supplementation on gene expression of insulin and lipid metabolism. We also did not measure vitamin E levels. In addition, further studies are necessary with single supplementation for each comparison with co-supplementation to assess the beneficial effects of each supplement on glycemic control and lipid profiles. Higher confidence interval of dependent variables found in some cases might make the findings difficult to be interpreted. Higher confidence interval might be explained by the small sample size in the included studies, which is another limitation of our study and thus more trials with larger sample size would be needed to confirm our findings.

## Conclusions

Overall, magnesium and vitamin E co-supplementation for 6 weeks in women with GDM significantly improved glycemic control and lipid profiles except for HDL-cholesterol levels.

## Abbreviations

FPG: fasting plasma glucose
HDL-cholesterol: high density lipoprotein-cholesterol
HOMA-IR: homeostasis model of assessment-insulin resistance
LDL-cholesterol: low density lipoprotein-cholesterol
QUICKI: quantitative insulin sensitivity check index
VLDL-cholesterol: very low density lipoprotein-cholesterol
